# Deficiency of mineralocorticoid receptor signalling in myeloid cells protects cardiac and kidney function in hypertensive diabetic mice

**DOI:** 10.1042/CS20256132

**Published:** 2025-12-19

**Authors:** Gregory H. Tesch, Elyce Ozols, James Morgan, Morag J. Young, David J. Nikolic-Paterson

**Affiliations:** 1 Monash Health (Department of Nephrology), Clayton, Victoria, Australia; 2 Monash University School of Clinical Sciences at Monash Health, Clayton, Victoria, Australia; 3 Monash University Centre for Inflammatory Diseases, Clayton, Victoria, Australia; 4 Hudson Institute of Medical Research, Clayton, Victoria, Australia; 5 Baker Heart and Diabetes Institute, Melbourne, Victoria, Australia; 6 Baker Department of Cardiometabolic Health and Disease, University of Melbourne, Victoria, Australia

**Keywords:** diabetic cardiomyopathy, diabetic nephropathy, fibrosis, macrophage, mineralocorticoid receptor

## Abstract

Mineralocorticoid receptor antagonists (MRAs) reduce hypertension, inflammation and tissue injury in human and experimental diabetes. Inflammation and injury in diabetic hearts and kidneys is also dependent on infiltrating macrophages. Therefore, we hypothesised that the tissue protective effects of MRAs in diabetes are dependent on mineralocorticoid receptor (MR) signalling in macrophages. To evaluate this hypothesis, transgenic mice with intact myeloid MR (*MR^flox/flox^*) or myeloid MR deficiency (*MR^flox/flox^ LysM^Cre^*) were developed on the hypertensive endothelial nitric oxide synthase deficient (*Nos3*-/-) mouse strain. Groups of these mice were made diabetic with streptozotocin and were assessed after 15 weeks for development of hypertension, cardiomyopathy and nephropathy. *Nos3*-/- mice with myeloid MR deficiency had equivalent diabetes and hypertension as myeloid MR intact controls but were protected against cardiac and renal function impairment. In diabetic hearts, myeloid MR deficiency reduced cardiomyocyte hypertrophy, capillary loss and fibrosis in association with reduced macrophage accumulation and a switch from an M1 to an M2 macrophage phenotype. In diabetic kidneys, myeloid MR deficiency reduced renal dysfunction (elevated plasma cystatin C) but did not protect against albuminuria, glomerulosclerosis or tubular damage; this was associated with a partial reduction in glomerular macrophages and an M1 to M2 macrophage phenotype switch. Therefore, MR signalling in macrophages promotes dysfunction in the diabetic heart and kidneys of *Nos3*-/- mice without affecting hypertension. Furthermore, abolishing macrophage MR signalling provides greater protection to hearts than kidneys during type 1 diabetes and hypertension, giving new insight into the mechanisms by which MRAs suppress tissue injury during diabetes.

## Introduction

Diabetes and hypertension are major risk factors for cardiovascular disease (CVD) and chronic kidney disease (CKD), which lead to organ failure and increased mortality [1,2]. Current therapies are primarily aimed at controlling blood glucose and hypertension but provide limited protection against cardiorenal complications and may not prevent progression of disease.

Extensive research has identified the mineralocorticoid receptor (MR) as an important therapeutic target for treating diabetic complications in preclinical models [3,4]. In addition, intervention with MR antagonists (spironolactone, eplerenone, finerenone) has been shown to protect diabetic patients from cardiorenal injury [5,6]. Studies in diabetic animal models have demonstrated that mineralocorticoid receptor antagonist (MRA) protection of the heart and kidney is partly due to reducing hypertension but also to reducing inflammation and fibrosis in these organs [5,6]. However, the use of MRAs is limited by their ability to alter the function of kidney tubules which induces hyperkalaemia [7]. The development of hyperkalaemia usually varies among patients, but if severe, can be life-threatening [7].

In order to avoid the clinical problem of hyperkalaemia, researchers have investigated the functional significance of MR in specific cell types that are critical to cardiorenal injury [8]. Gene deletion studies have shown that MR signalling in myeloid cells reduces inflammation and fibrosis in non-diabetic mouse models of cardiac or kidney disease [9–12], suggesting that this could also be an important pathological pathway in diabetic cardiorenal disease.

Genetic deletion of MR in myeloid cells has been shown to alter the phenotype of macrophages by reducing M1 activation and favouring alternative M2 activation [10,13,14]. This phenotype switching can reduce macrophage recruitment and proinflammatory responses within tissues, which can suppress injury and subsequent development of fibrosis. These pathological processes are also critical for injury in diabetic hearts and kidneys.

In the current study, we have created novel double transgenic mice to explore the pathological importance of macrophage MR signalling in a model of diabetic cardiac and kidney disease exacerbated by hypertension. Mice genetically deficient in myeloid MR were crossed onto mice genetically deficient in *Nos3* which spontaneously develop hypertension, have increased susceptibility to diabetic tissue injury and display more rapid progression of cardiac and kidney disease [15,16]. This model was designed to dissect the role of macrophage MR signalling in the development of inflammation, fibrosis and organ function decline in the setting of diabetes and moderate hypertension.

## Materials and methods

### Myeloid MR-deficient mice

Conditional gene deletion of the MR was performed in C57BL/6 mice using the CreLoxP system. Mice with homozygous floxed MR gene were crossed with littermates expressing Cre recombinase under the control of the Lysozyme M promoter [9] to create *MR^flox/flox^ LysM^Cre/-^* mice lacking MR in mature myeloid cells (neutrophils, monocytes and macrophages). Previous characterisation of *MR^flox/flox^ LysM^Cre/-^* mice has shown that they have normal structure and function in hearts and kidneys [9,12]. In the current study, *MR^flox/flox^ LysM^Cre/-^* and myeloid *MR^flox/flox^* wildtype littermates were crossed with mice lacking endothelial nitric oxide synthase (*Nos3^-/-^*) which are spontaneously hypertensive and have increased susceptibility to diabetic tissue injury. The resulting *Nos3^-/-^ MR^flox/flox^* mice (called *MR^WT^*) were used as controls in experimental studies and *Nos3^-/-^ MR^flox/flox^ LysM^cre/-^* mice (called *MR^My^*) were used to identify the impact of myeloid MR deficiency on the development of cardiac and kidney injury after the development of diabetes. The genotyping details are presented in Supplementary Table S1.

### Animal model of diabetes

Only male mice of the selected genotypes were used in this study because they are more susceptible to developing a stable type 1 diabetes after receiving streptozotocin [17] and developing cardiac disease [15]. At nine weeks of age, male *MR^WT^* and *MR^My^* mice were given intraperitoneal injections of streptozotocin (STZ; 5 × 55 mg/kg/day; Sigma, St Louis, MO, U.S.A.). Groups of mice (*n* = 13) with diabetes (fasting blood glucose > 16 mmol/l at two weeks after the last STZ injection) were maintained on a conventional diet under standard animal house for 15 weeks. Age-matched non-diabetic mice (*n* = 10) were used as controls. Blood glucose (measured by a tail vein sample) and body weight were measured weekly after a 3 h fast (0800–1100) and mice with fasting glucose levels > 30 mmol/l were given 0.5 units of protophane insulin (Novo Nordisk, Sydney, Australia) subcutaneously three times a week to maintain body weight. Urine was collected at week 15 after STZ injection to assess urine albumin excretion. Glycated haemoglobin (%HbA_1_c) was measured from blood samples taken at week 15. Hearts and kidneys were collected at week 15, cut into cross-sections and were fixed in 4% (vol/vol) formaldehyde, 2% (wt/vol) paraformaldehyde–lysine–periodate (PLP) or snap-frozen and stored at −80°C. These animal studies were performed at the Monash Medical Centre Animal Facility and were approved by the Monash Medical Centre Animal Ethics Committee (approval MMCB/2015/26) in accordance with the Australian Code of Practice for the Care and Use of Animals for Scientific Purposes, 8^th^ edition (2013).

### Biochemistry

Fasting blood glucose was measured from tail blood by glucometer (Medisense, Abbott Laboratories, Bedford, MA, U.S.A.). Urine was collected from mice housed in metabolic cages for 6 h. Heparinised whole blood was collected from tail veins for analysis of HbA_1_c. At the end of experimentation, whole blood was collected by cardiac puncture in anaesthetised animals and stored as serum or heparinised plasma. Urine creatinine levels were determined by the Jaffe rate reaction method. ELISA kits were used to assess levels of urine albumin (Cat# E90-134, Bethyl Laboratories, Montgomery, TX, U.S.A.) and serum cystatin-C (Cat# DY1238, R&D systems, Minneapolis, MN, U.S.A.). HbA_1_c was measured by DCA Vantage Analyzer (Siemens, Camberley, U.K.).

### Blood pressure analysis

Systolic blood pressure (SBP) was measured in conscious mice by tail-cuff plethysmography (IITC Life Science, Woodland Hills, CA, U.S.A.). Mice were trained twice weekly for three weeks prior to experimental readings at 15 weeks after the final STZ dose or in age-matched non-diabetic controls. At each recording, mice were acclimatised to a preheated chamber (29°C) for 15 min and the pressure readings were recorded over three consecutive manual inflation–deflation cycles to obtain an average.

### Echocardiography

We measured left ventricular (LV) function and dimensions by transthoracic two-dimensional echocardiography using a 13-MHz linear transducer (GE Healthcare, Chicago, IL, U.S.A.) at a sweep speed of 100 mm/sec. Mice were sedated on 4% isoflurane and anaesthesia was maintained by a mixture of O_2_ and 2.5% isoflurane. Structural cardiac parameters were measured on M-mode recordings from at least three cardiac cycles. M-mode tracing in parasternal short axis view at the height of the papillary muscle was used to measure LV internal diameter at end systole and end diastole. Fractional shortening (FS) was calculated from these internal diameters using the following formula: FS = ((LV end diastolic diameter – LV end systolic diameter) / LV end diastolic diameter) × 100%.

### Antibodies

The primary antibodies used in this study were: rat anti-mouse CD68 (FA-11; Cat# MCA1957, Serotec, Oxford, U.K.); goat anti-collagen IV (Southern Biotechnology, Birmingham, AL, U.S.A.); rabbit anti-mouse collagen 1α1 (E8F4L XP, Cat# 72026, Cat 1340–01, Cell Signaling Technology, Danvers, MA, U.S.A.); and rabbit anti-mouse CD31 (D8V9E XP, Cat# 77699, Cell Signaling Technology).

### Immunohistochemistry

Formalin-fixed sections (2 or 4 μm) were stained with Periodic acid-Schiff (PAS) reagent to assess structure and counterstained with haematoxylin to identify nuclei. Immunostaining of collagen 1 and CD31 was performed on 4 μm formalin-fixed sections from paraffin-embedded tissues following heat antigen retrieval (95C, 40 min) at pH 9 (Tris-EDTA buffer) or pH 6 (sodium-citrate buffer), respectively. Immunostaining of collagen IV was performed on 4 μm methylcarn-fixed sections from paraffin-embedded tissues. Immunostaining for CD68 was performed on 5 μm cryostat sections from tissues fixed in PLP solution. For immunostaining, sections were treated with 20% rabbit serum or 20% goat serum for 30 min and then incubated with primary antibody in 3% BSA overnight at 4°C. Sections were then placed in 0.6% hydrogen peroxide in methanol for 20 min to inactivate endogenous peroxidase. Bound primary antibodies were detected using a standard ABC–peroxidase system: avidin–biotin block, biotinylated antibodies (rabbit anti-goat IgG, rabbit anti-rat IgG or goat anti-rabbit IgG) and ABC-peroxidase (Vector Laboratories, Burlingame, CA, U.S.A.). Sections were developed with 3,3-diaminobenzidine (Sigma) to produce a brown colour. Sections with CD68 labelling were counterstained with PAS and haematoxylin to assist cell counting. Normal goat serum or isotype-matched irrelevant IgG was used as negative controls.

### Analysis of histochemistry and immunohistochemistry

Immunohistochemistry images were obtained using an Olympus BX43 microscope and an Olympus DP27 camera, and were analysed with Olympus CellSens Standard 1.18 software (Olympus LifeSciences, Japan). Cardiomyocyte volume was assessed in a minimum of 100 myocytes/heart from five to six fields (magnification ×400) on sections stained with haematoxylin and PAS reagent, using computer morphometric analysis. Cardiac collagen was identified with 0.1% Sirius Red in saturated picric acid (Sigma-Aldrich, Castlehill, Australia). Cardiac collagen I, cardiac CD31 and glomerular collagen IV were identified by immunostaining. All collagen and CD31 staining was assessed on blinded slides by computer image analysis. Cardiac interstitial collagen content was quantified as a percentage of total myocardial area stained (magnification ×200), excluding blood vessels. Cardiac CD31 was assessed as the number of capillaries stained per mm^2^ from five to six fields (magnification ×400). Glomerular collagen IV was quantified as the percentage of glomerular area stained (magnification ×400) in 25 hilar glomerular cross-sections per kidney. Cardiac and kidney macrophage accumulation was determined by immunostaining of CD68. Cardiac and kidney interstitial macrophages were assessed as CD68+cells/mm^2^ in tissue cross-sections (magnification ×200). Glomerular macrophages were determined by counting CD68+cells/glomerulus in 20 hilar glomerular cross-sections (gcs) per animal (magnification ×400). All scoring was performed on blinded slides.

### Real-time PCR

Total RNA was extracted from whole heart or kidney using Trizol (Invitrogen) and reverse transcribed with random primers using the Superscript III First-Strand Synthesis kit (Invitrogen). Real-time PCR analysis was performed as previously described using FAM-labelled Taqman target probes and VIC-labelled 18S probe as a reference [12]. All gene expression data are presented with the MR-WT group expression set to 1. The Taqman gene expression assays (Applied Biosystems, Waltham, MA, U.S.A.) are listed in Supplementary Table S2.

### Statistical analysis

Statistical differences were analysed by either the unpaired Student’s *t*-test or two-way ANOVA with Tukey’s HSD multiple comparison post-test. Data were recorded as mean ± SD with *P*<0.05 considered significant. All analyses were performed using GraphPad Prism 8.0 (GraphPad software, San Diego, CA, U.S.A.).

## Results

### Myeloid MR deficiency does not affect development of diabetes in Nos3-/- mice

**Figure 1:**
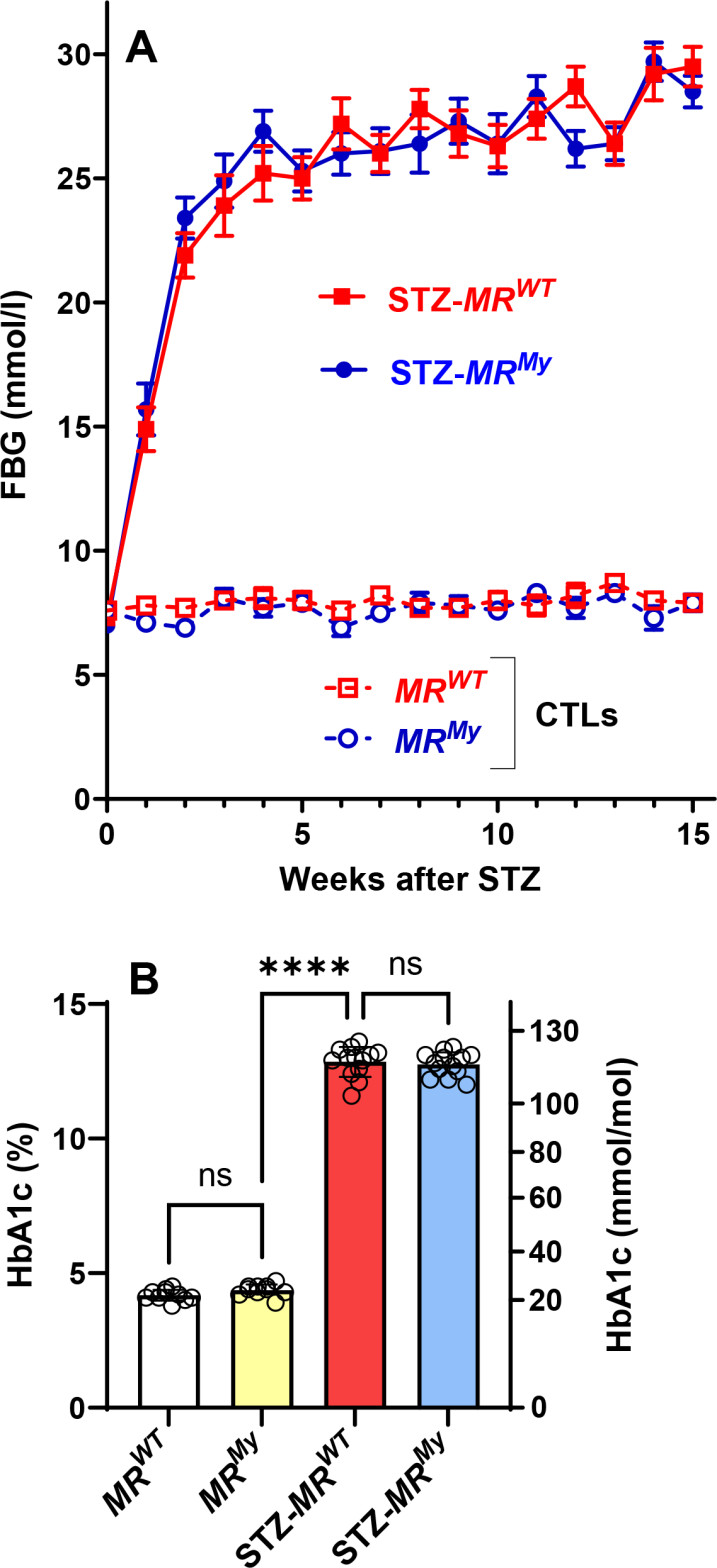
Myeloid MR signalling does not affect the development of type 1 diabetes. Groups of hypertensive *Nos3-/-* male mice with either intact myeloid MR signalling (*MR^WT^*) or myeloid MR deficiency (*MR^My^*) were given streptozotocin (STZ) to induce type 1 diabetes. The development of diabetes was monitored for 15 weeks after receiving STZ. Age-matched non-diabetic mice (ND), with or without myeloid MR signalling, were used as controls. The development of diabetes was monitored by measurements of (**A**) fasting blood glucose (FBG) performed weekly and (**B**) the accumulation of glycated haemoglobin (HbA_1_c levels) in blood at week 15 after STZ. Data = mean ± SEM in (**A**) and individual data points with mean ± SD in (**B**). *n* = 10–13. ****P<0.0001, ns = non-significant.

Non-diabetic *MR^WT^* and *MR^My^* mice exhibited normal fasting blood glucose levels throughout the experiment. In comparison, diabetic *MR^WT^* and *MR^My^* mice showed a similar rapid rise in fasting blood glucose levels during the first 2 weeks after streptozotocin, which reached a plateau by week 4 (Figure 1A). The development of diabetes resulted in a three-fold increase in the levels of glycated haemoglobin (HbA_1_c) at week 15, which was equivalent in *MR^WT^* and *MR^My^* mice (Figure 1B).

### Myeloid MR deficiency protects against cardiac and renal function impairment without reducing hypertension or albuminuria in diabetic Nos3-/- mice

**Figure 2:**
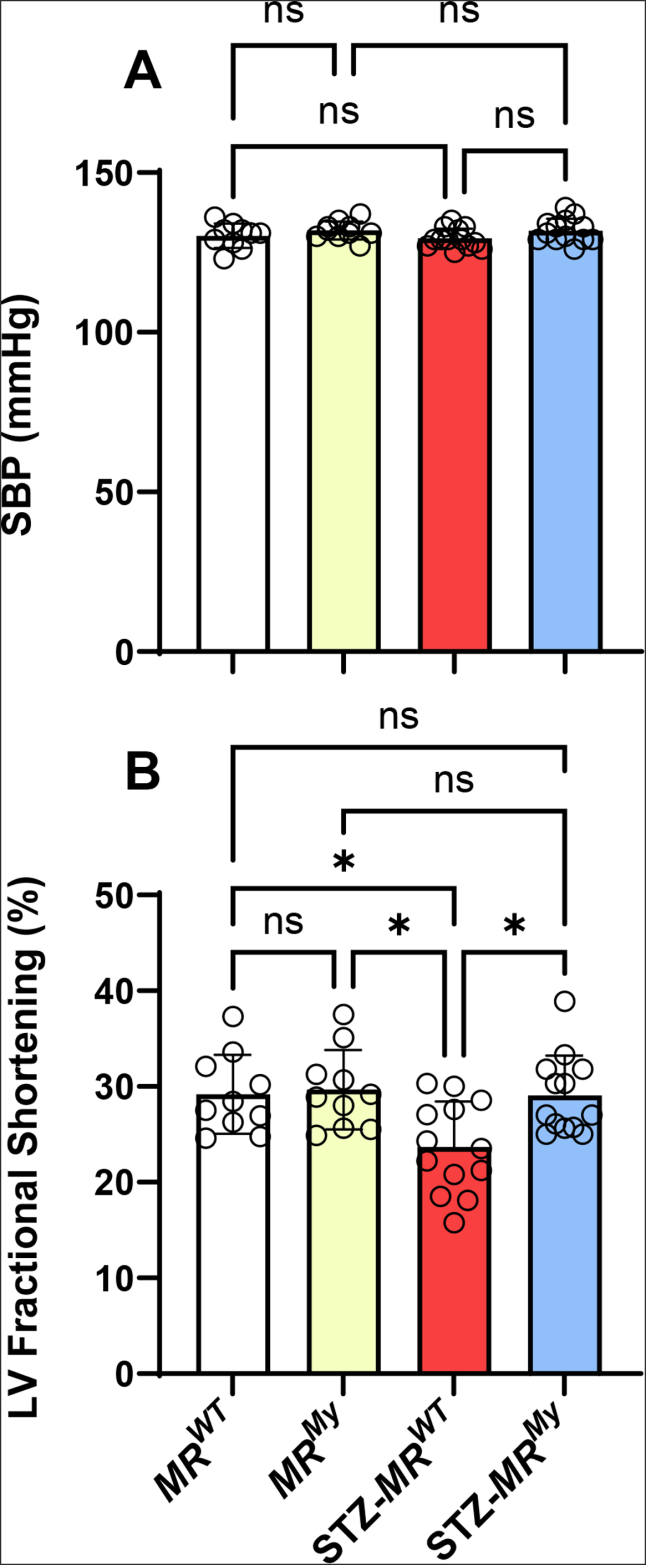
Deficiency of myeloid MR signalling during diabetes prevents cardiac dysfunction without reducing hypertension. (**A**) *MR^WT^* mice displayed mild hypertension, which was unaffected by the development of diabetes, or the absence of myeloid MR signalling. (**B**) Compared with non-diabetic controls, diabetic *MR^WT^* mice were found to have left ventricular fractional shortening, which was prevented in diabetic *MR^My^* mice. Data = individual data points with mean ± SD. *n* = 10–13. *P < 0.05, ns = non-significant.

At week 15 of experimentation, non-diabetic *MR^WT^* and *MR^My^* mice displayed moderate hypertension with a systolic blood pressure (SBP) of 130 ± 4 mmHg. Hypertension was unaffected by the development of diabetes or the absence of myeloid MR signalling (Figure 2A). Similarly, deficiency of myeloid MR had no impact on cardiac function in non-diabetic mice. However, the development of diabetes in *MR^WT^* mice reduced left ventricular end diastolic diameter and fractional shortening, resulting in impairment of left ventricular function (indicated by a 19% reduction in left ventricular volume), which was prevented in diabetic *MR^My^* mice (Figure 2B, Supplementary Table S3).

**Figure 3:**
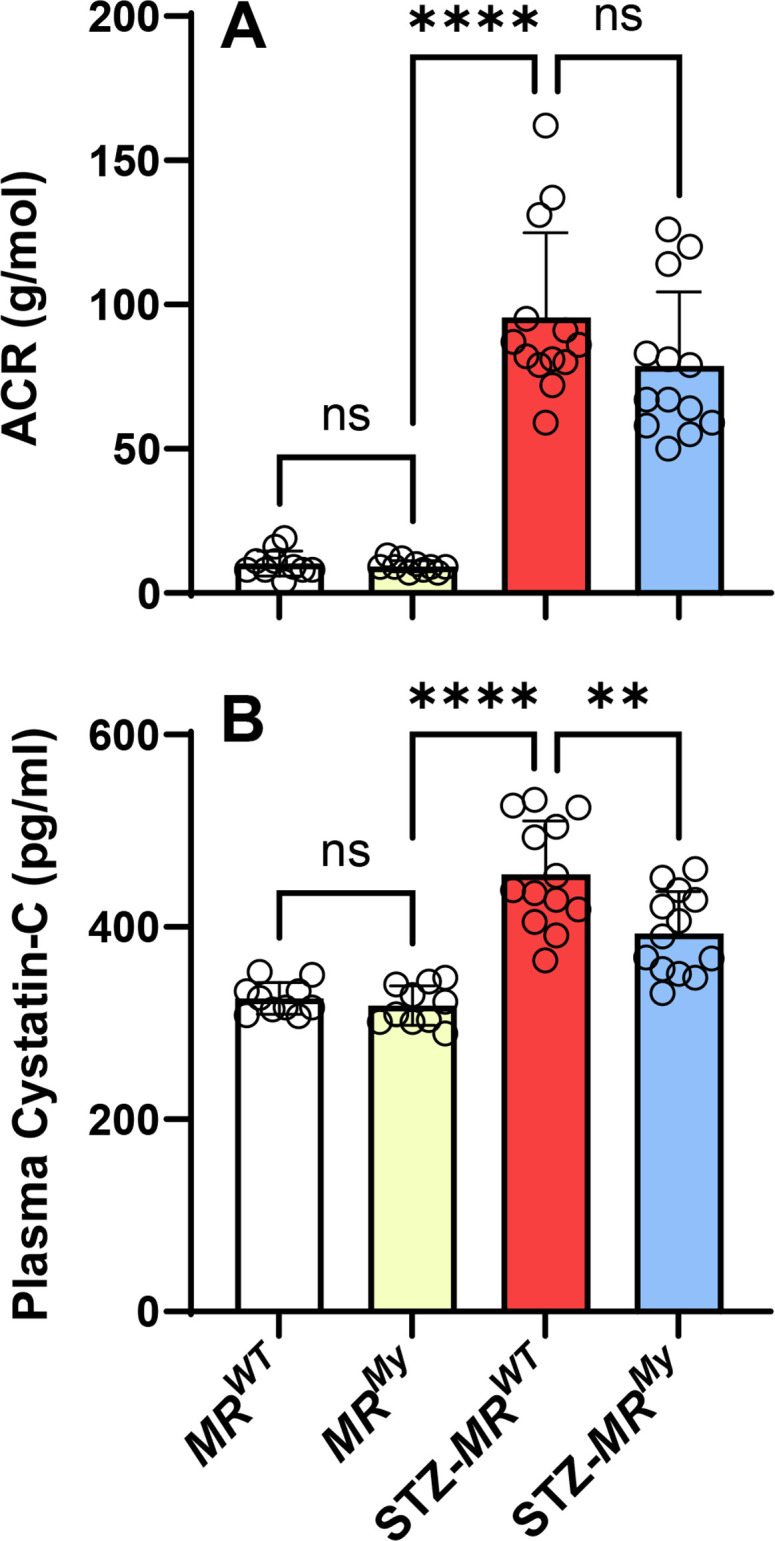
Deficiency of myeloid MR signalling during diabetes inhibits kidney dysfunction without reducing albuminuria. (**A**) Development of diabetes induced a marked increase in the urine albumin:creatinine ratio (ACR), which was similar in *MR^WT^* and *MR^My^* mice. (**B**) Compared with non-diabetic controls, diabetes induced an increase in plasma cystatin-C levels in *MR^WT^* mice indicating mild renal dysfunction, which was reduced in *MR^My^* mice. Data = individual data points with mean ± SD. *n* = 10–13. ****P<0.0001, **P<0.01, ns = non-significant.

In non-diabetic *MR^WT^* and *MR^My^* mice, levels of urine albumin excretion (albumin: creatinine ratio, ACR) and plasma levels of cystatin-C were normal (Figure 3). After 15 weeks of diabetes, urine albumin excretion increased approximately ten-fold in *MR^WT^* and *MR^My^* mice (Figure 3A). Diabetes also increased plasma levels of cystatin-C by 40% in *MR^WT^* mice, indicating significant renal dysfunction (Figure 3B). However, diabetic *MR^My^* mice were partially protected from this renal dysfunction, showing a 41% reduction in plasma cystatin-C levels (Figure 3B).

### Myeloid MR deficiency reduces cardiac and renal macrophages and promotes alternative macrophage activation in diabetic Nos3-/- mice

**Figure 4:**
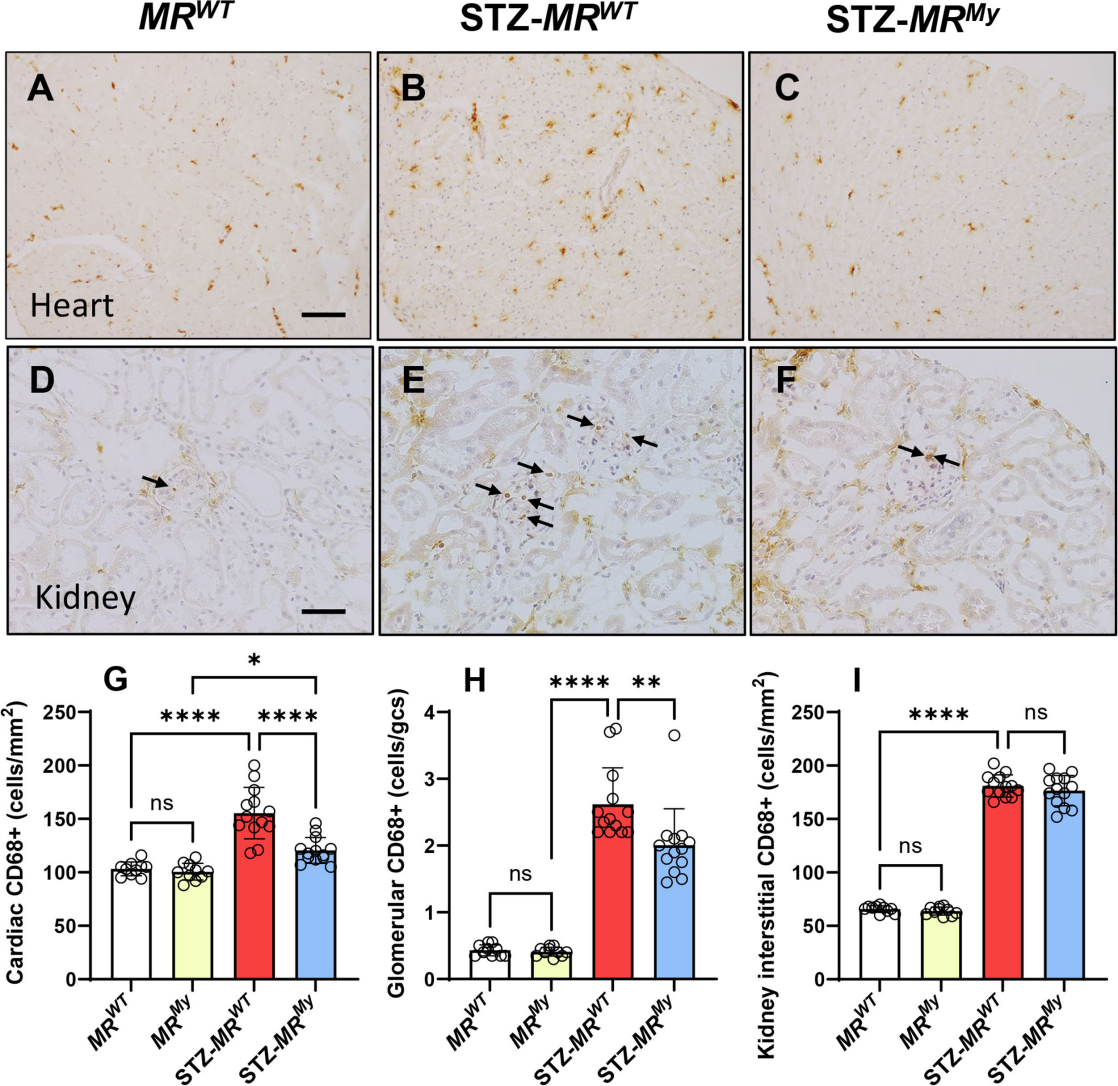
Deficiency of myeloid MR signalling affects macrophage accumulation in diabetic tissues. Immunostaining of CD68 (brown) with weak hematoxylin stained nuclei (blue) shows resident macrophages in (**A**) a heart and (**D**) a kidney glomerulus (arrow) of a non-diabetic mouse. In comparison, diabetes-induced inflammation results in increased accumulation of CD68+macrophages in (**B**) a heart and (**E**) a glomerulus of a *MR^WT^* mouse, which is reduced in (**C**) a heart and (**F**) a glomerulus (arrows) of a *MR^My^* mouse. Quantification of CD68+macrophage accumulation is shown graphically for (**G**) the hearts, (**H**) the kidney glomeruli, and (**I**) the kidney interstitium of *MR^WT^* and *MR^My^* mice with and without diabetes. Images: Bar (**A,C,E**) = 100 µm; (**B,D,F**) = 50 µm. Graph displays individual data points with mean ± SD, *N* = 10–13. ****P<0.0001, **P<0.01, *P<0.05, ns = non-significant.

Immunostaining of CD68 identified a substantial infiltrate of macrophages in the hearts of diabetic *MR^WT^* mice which increased by 50% compared with non-diabetic controls (Figure 4A, B and G). This increase in cardiac macrophages was reduced by 66% in diabetic *MR^My^* mice (Figure 4C and G). In comparison, diabetes had a more diverse effect on cardiac gene expression of inflammatory cell markers. Cardiac gene expression of *Cd68* (macrosialin) was 29% lower and *Adgre1* (F4/80 antigen) was 33% elevated, while *Elane* (neutrophil elastase) was unchanged in diabetic hearts compared with non-diabetic *MR^WT^* mice. These effects did not differ in diabetic *MR^My^* mice (Table 1).

**Table 1:** Cardiac gene expression data not displayed in graphs

*Gene expression* *(mRNA/18 s)*	*MR^WT^*	*MR^My^*	*STZ-Diabetes*	
			*MR^WT^*	*MR^My^*
*Cd68*	1.00 ± 0.18	1.03 ± 0.17	0.71 ± 0.16^B^	0.67 ± 0.10^B^
*Adgre1*	1.00 ± 0.15	0.97 ± 0.20	1.32 ± 0.34^a^	1.17 ± 0.28
*Elane*	1.00 ± 0.21	1.00 ± 0.29	1.00 ± 0.32	0.91 ± 0.34
*Tnf*	1.00 ± 0.20	1.01 ± 0.26	1.12 ± 0.28	0.97 ± 0.22
*Ccl2*	1.00 ± 0.23	1.03 ± 0.17	0.54 ± 0.19^B^	0.58 ± 0.16^B^
*Nox4*	1.00 ± 0.22	1.01 ± 0.22	1.14 ± 0.37	1.13 ± 0.37
*Nlrp3*	1.00 ± 0.16	1.02 ± 0.23	0.86 ± 0.33	1.05 ± 0.36
*Tgfb1*	1.00 ± 0.21	0.94 ± 0.19	1.63 ± 0.25^B^	1.34 ± 0.35
*Timp1*	1.00 ± 0.21	1.01 ± 0.18	1.52 ± 85	1.69 ± 0.79
*Col1α1*	1.00 ± 0.19	1.01 ± 0.20	0.78 ± 0.12^a^	0.85 ± 0.17
*Col3α1*	1.00 ± 0.19	1.02 ± 0.17	0.85 ± 0.36	0.93 ± 0.19
*Fn1*	1.00 ± 0.28	0.97 ± 0.24	0.93 ± 0.35	1.03 ± 0.43
*Cdkn1a*	1.00 ± 0.21	1.02 ± 0.20	0.91 ± 0.21	1.05 ± 0.34
*Cdkn2a*	1.00 ± 0.22	0.96 ± 0.27	0.93 ± 0.30	1.09 ± 0.33

Data = mean ± standard deviation. *n *= 10–13. ^a^ *P*<0.05, ^ b^ *P*<0.0001 vs non-diabetic control.

Inflammation was more profound in the kidneys of diabetic *MR^WT^* mice, where the number of CD68+macrophages increased by 600% in kidney glomeruli and 270% in the renal interstitium compared with non-diabetic controls (Figure 4D, H and I). In diabetic *MR^My^* mice, the accumulation of CD68+macrophages was reduced by 28% in glomeruli but was not reduced in the kidney tubulointerstitium (Figure 4F, H and I). Gene expression of *Cd68, Adgre1* and *Elane* was also substantially increased in diabetic kidneys (Table 2) but was unaffected by myeloid MR deficiency, suggesting that the accumulation of macrophages and neutrophils in whole kidney was not significantly influenced by myeloid MR signalling.

**Table 2:** Kidney gene expression data not displayed in graphs

Gene expression (mRNA/18 s)	MR^WT^	MR^My^	STZ-Diabetes	
			MR^WT^	MR^My^
Cd68	1.0 ± 0.2	1.1 ± 0.2	2.8 ± 0.5^b^	2.9 ± 0.5^b^
Adgre1	1.0 ± 0.2	1.1 ± 0.3	2.1 ± 0.5^b^	1.9 ± 0.3^b^
Elane	1.0 ± 0.4	1.1 ± 0.4	4.4 ± 1.2^b^	4.4 ± 1.4^b^
Tnf	1.0 ± 0.2	1.0 ± 0.2	3.1 ± 0.8^b^	3.5 ± 0.9^b^
Ccl2	1.0 ± 0.2	1.0 ± 0.2	4.1 ± 1.1^b^	4.2 ± 1.2^b^
Nos2	1.0 ± 0.1	1.0 ± 0.2	1.8 ± 0.3^b^	1.8 ± 0.3^b^
Nlrp3	1.0 ± 0.2	1.1 ± 0.1	2.4 ± 0.4^b^	2.5 ± 0.4^b^
Vegfa	1.0 ± 0.2	1.0 ± 0.2	1.2 ± 0.1	1.8 ± 0.5^b,c^
Pdgfb	1.0 ± 0.2	1.1 ± 0.2	1.2 ± 0.3	1.3 ± 0.2
Ctgf/Ccn2	1.0 ± 0.3	1.1 ± 0.2	2.3 ± 0.6^b^	2.4 ± 0.6^b^
Tgfb1	1.0 ± 0.2	1.2 ± 0.2	2.0 ± 0.5^a^	2.1 ± 0.6^b^
Timp1	1.0 ± 0.2	1.2 ± 0.2	4.9 ± 2.0^b^	4.3 ± 1.6^b^
Col1α1	1.0 ± 0.2	1.2 ± 0.3	4.7 ± 1.0^b^	4.5 ± 1.4^b^
Col4α1	1.0 ± 0.2	1.1 ± 0.3	3.2 ± 0.8^b^	3.4 ± 0.7^b^
Fn1	1.0 ± 0.2	1.1 ± 0.2	5.9 ± 1.0^b^	6.5 ± 1.5^b^
Kim1	1.0 ± 0.3	1.1 ± 0.4	9.7 ± 2.6^b^	8.8 ± 2.4^b^
Cdkn1a	1.0 ± 0.3	1.2 ± 0.3	18.0 ± 5.5^b^	17.8 ± 3.8^b^
Cdkn2a	1.0 ± 0.3	1.2 ± 0.3	10.2 ± 2.6^b^	9.6 ± 2.0^b^

Data = mean ± standard deviation. *n* = 10–13. ^a^ *P*<0.001, ^b^ *P*<0.0001 vs non-diabetic control. ^c^ *P*<0.0001 vs MyMRWT with diabetes.

**Figure 5:**
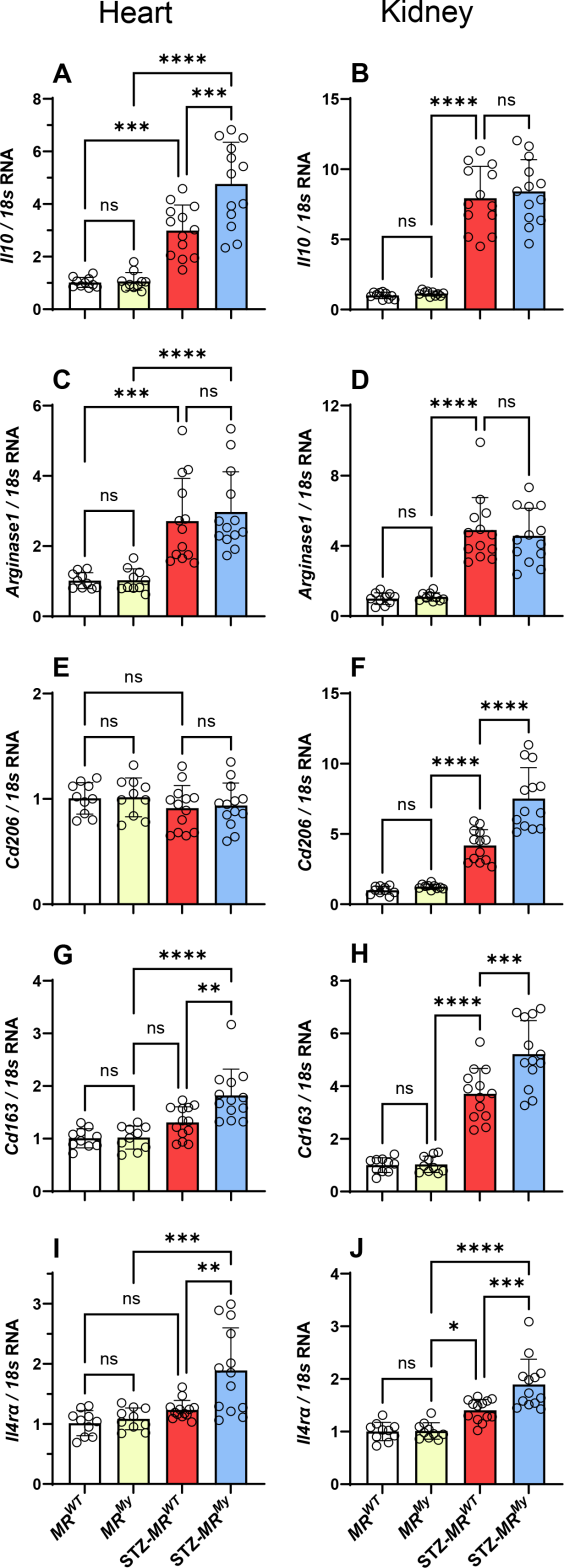
Deficiency of myeloid MR signalling promotes a M2 macrophage phenotype in diabetic tissues. Gene expression analysis of diabetic *MR^WT^* hearts found increased mRNA levels of (**A**) *Il10* and (**C**) *Arginase1* but no increase in mRNA levels of (**E**) *Cd206,* (**G**) *Cd163* and (**I**) *Il4ra*. Myeloid MR deficiency had no impact on the mRNA levels of *Arginase1* or *Cd206* in diabetic hearts but did increase the mRNA levels of *Il10, Cd163* and *Il4ra*. In comparison, diabetic *MR^WT^* kidneys exhibited increased expression of (**B**) *Il10* and (**D**) *Arginase1*, (**F**) *Cd206,* (**H**) *Cd163* and (**J**) *Il4ra*. Myeloid MR deficiency had no impact on the mRNA levels of *Il10* or *Arginase1* in diabetic kidneys but did further increase the mRNA levels of *Cd206*, *Cd163* and *IL4ra*. Graph displays individual data points with mean ± SD, *n* = 10–13. ****P<0.0001, ***P<0.001, **P<0.01, *P<0.05, ns = non-significant.

While the accumulation of macrophages in diabetic kidneys was accompanied with an increased expression of proinflammatory genes (*Tnf, Ccl2, Nos2, Nlrp3* – Table 1) associated with classical M1 macrophage activation, this was not affected by myeloid MR deficiency and was not seen in the diabetic hearts (Table 2). Surprisingly, in diabetic hearts, gene expression of *Ccl2* was decreased and *Tnf* gene expression remained similar to non-diabetic hearts (Table 2), while *Il10* expression increased (Figure 5), suggesting a shift towards alternate M2 activation. In addition, gene expression analysis identified an increase of M2 macrophage markers in the hearts (*Il10*, *Il4ra, Cd163*) and kidneys (*Il4ra, Cd163, Cd206*) of diabetic *MR^My^* mice compared with diabetic *MR^WT^* mice (Figure 5), indicating that myeloid MR deficiency promotes an M2 macrophage phenotype in these diabetic tissues.

### Myeloid MR deficiency reduces injury and fibrosis in the hearts, but not kidneys, of hypertensive Nos3-/- diabetic mice

**Figure 6:**
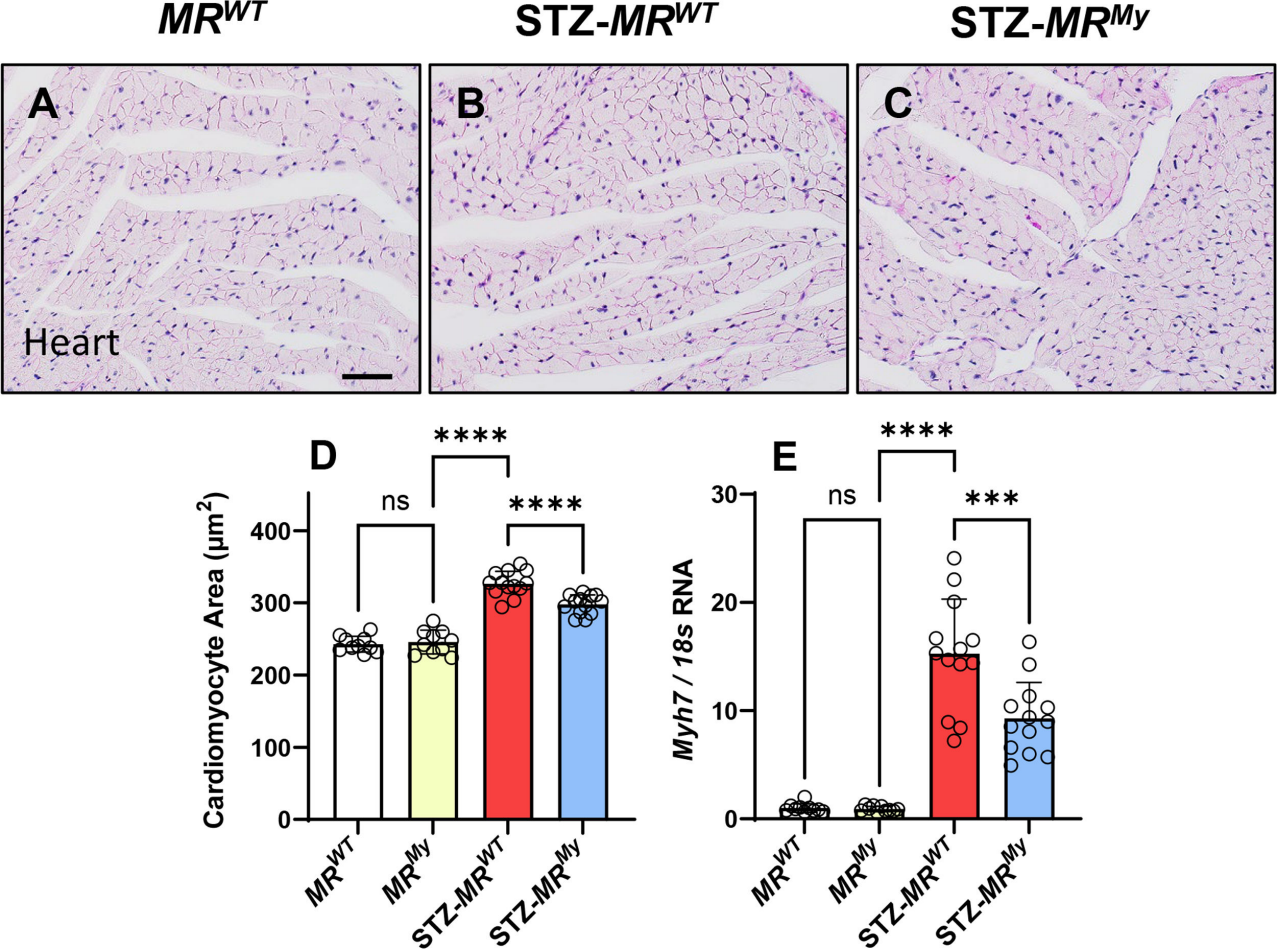
Deficiency of myeloid MR signalling reduces myocyte hypertrophy in diabetic hearts. Histology staining with periodic acid Schiff’s (PAS) reagent and hematoxylin shows cardiomyocytes in (**A**) a non-diabetic *MR^WT^* mouse heart, (**B**) a diabetic *MR^WT^* mouse heart, and (**C**) a diabetic *MR^My^* mouse heart. (**D**) Graphed morphometric analysis shows an increase in cardiomyocyte area in *MR^WT^* mice with diabetes compared with non-diabetic mice, which is reduced in *MR^My^* mice with diabetes. (**E**) Cardiac analysis also identified increased *Myh7* gene expression (a marker of in cardiomyocyte hypertrophy) in diabetic *MR^WT^* mice, which was reduced in diabetic *MR^My^* mice. Images: Bar (**A-C**) = 50 µm. Graph displays individual data points with mean ± SD, *n* = 10–13. ****P<0.0001, ***P<0.001, ns = non-significant.

At week 15 of diabetes, morphometric analysis identified a 35% increase in cardiomyocyte size in *MR^WT^* mice which coincided with increased cardiac gene expression of the hypertrophy marker *Myh7* (15-fold) that encodes beta-myosin heavy chain (Figure 6). Deficiency of myeloid MR in diabetic hearts reduced the increase in myocyte size by 35% and inhibited elevated gene expressions of *Myh7* by 40%, indicating suppression of cardiac hypertrophy (Figure 6).

**Figure 7:**
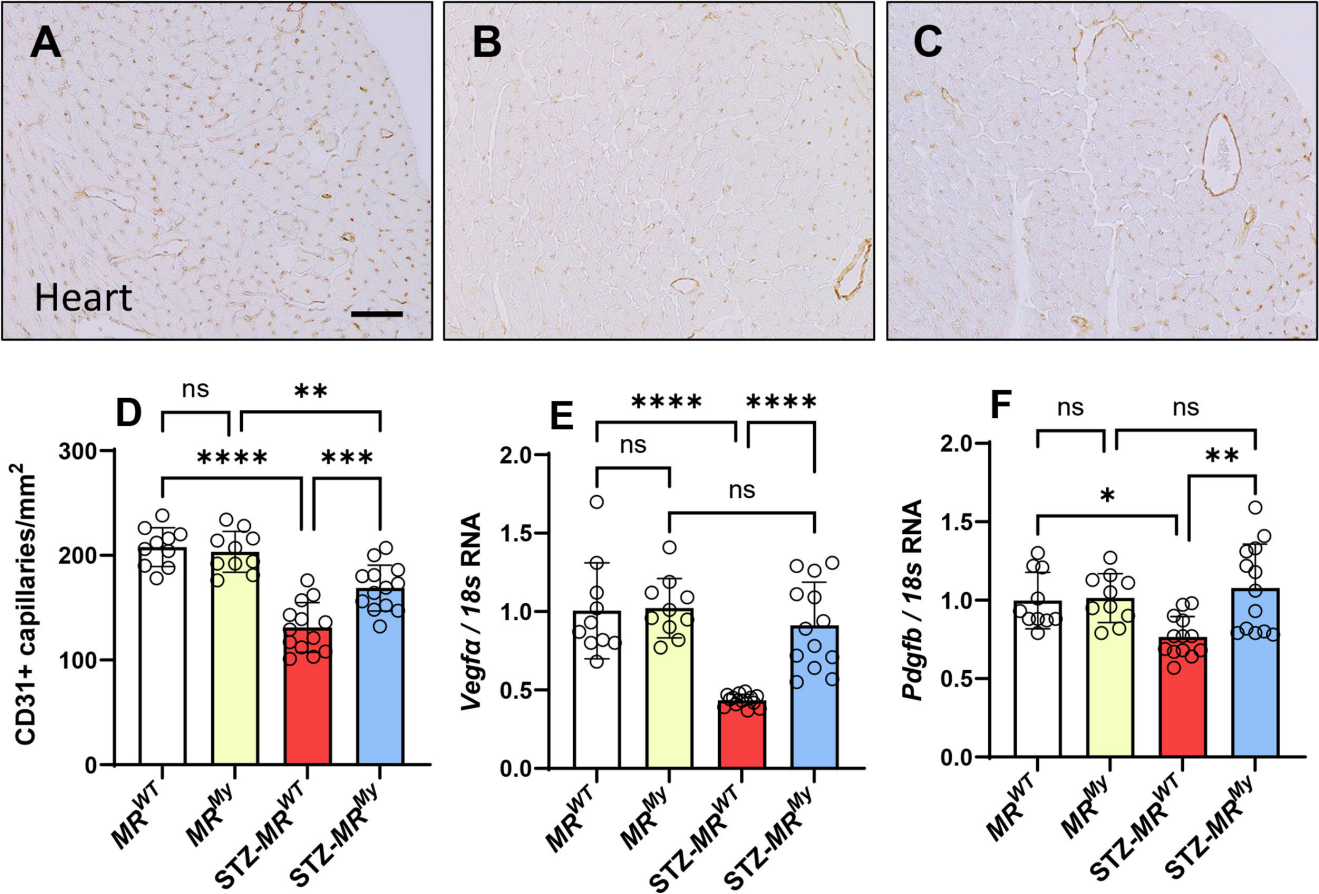
Deficiency of myeloid MR signalling reduces capillary loss in diabetic hearts. Immunostaining of CD31 (brown) shows capillary cross-sections adjacent to myocytes in (**A**) a non-diabetic *MR^WT^* mouse heart. In comparison, CD31 immunostaining is weaker and identifies less capillaries in (**B**) a diabetic *MR^WT^* mouse heart. In contrast, CD31 immunostaining is stronger and detects more capillaries in (**C**) a diabetic *MR^My^* mouse. (**D**) Graphed quantitative analysis shows that diabetes induces a loss of CD31 + cardiac capillaries in *MR^WT^* mice, which is protected in *MR^My^* mice. Graphs of cardiac gene expression show that diabetes induces reductions in (**E**) *Vegfa* and (**F**) *Pdgfb* in the hearts of *MR^WT^* mice which is absent in *MR^My^* mice. Images: Bar (**A-C**) = 50 µm. Graph displays individual data points with mean ± SD, *n* = 10–13. ****P<0.0001, ***P<0.001, **P<0.01, *P<0.05, ns = non-significant.

Diabetes also induced vascular changes in diabetic hearts. At week 15 of diabetes, analysis of CD31 immunostaining identified a loss of cardiac capillary density of 37% in *MR^WT^* mice, which corresponded with a 57% reduction in gene expression of *Vegfa* and a 23% reduction in *Pdgfb* (Figure 7). In comparison, deficiency of myeloid MR reduced the loss of capillary density by 18% and prevented the diabetes-induced reductions in *Vegfa* and *Pdgfb* (Figure 7), thereby partially protecting mice from diabetes-induced vascular abnormalities in the heart.

**Figure 8:**
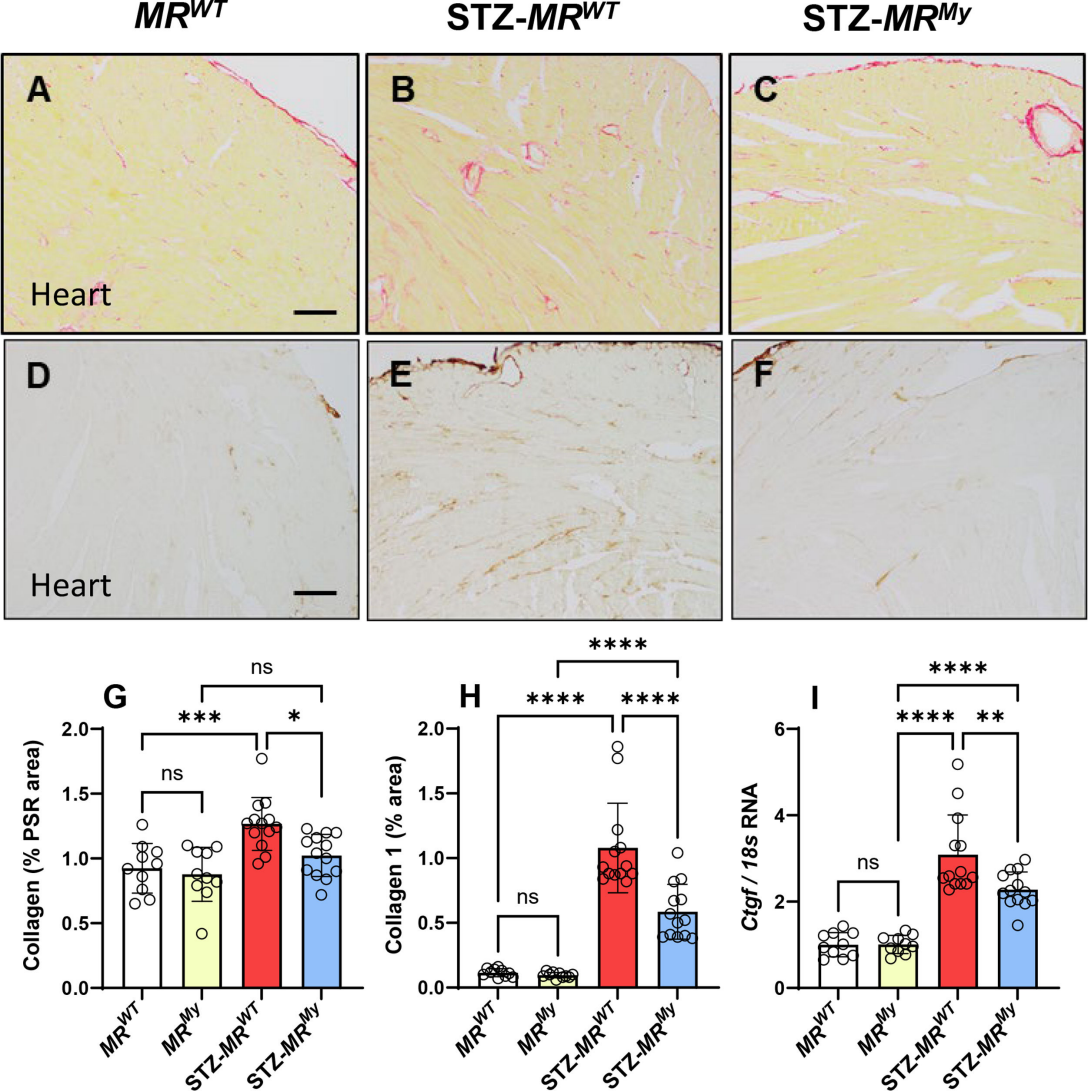
Deficiency of myeloid MR signalling inhibits cardiac fibrosis during diabetes. Histological staining with picrosirius red (pink) shows some collagen in vessels in (**A**) a non-diabetic *MR^WT^* mouse heart, and increased collagen deposition in (**B**) a diabetic *MR^WT^* mouse heart, which is reduced in (**C**) a diabetic *MR^My^* mouse heart. Similarly, immunostaining shows very little deposition of collagen 1 (brown) in (**D**) a non-diabetic *MR^WT^* mouse heart, which is substantially increased in (**E**) a diabetic *MR^WT^* mouse heart, but reduced in (**F**) a diabetic *MR^My^* mouse heart. Graphed analysis of (**G**) the picrosirius red stained area, (**H**) the immunostained area of collagen 1, and (**I**) cardiac gene expression of *ctgf* shows that diabetes increases collagen deposition and *ctgf* mRNA in the hearts of *MR^WT^* mice but is reduced in *MR^My^* mice. Images: Bar (**A-F**) = 100 µm. Graphs display individual data points with mean ± SD, *n* = 10–13. ****P<0.0001, ***P<0.001, **P<0.01, *P<0.05, ns = non-significant.

To further understand the impact of myeloid MR signalling on diabetic hearts, we assessed the accumulation of collagen and expression of fibrotic genes. At week 15 of diabetes, histological staining of hearts with picrosirius red (PSR) demonstrated a 50% increase in the deposition of total cardiac collagen in diabetic *MR^WT^* mice, and immunostaining of collagen-1α1 found a 10-fold increase in cardiac collagen 1 deposition in these mice (Figure 8). There was increased cardiac gene expression of the fibrotic markers *Tgfb1*, *Ctgf/Ccn2, Pai1/Serpine1* and *Timp1* in diabetic *MR^WT^* mice, but no increase in gene expression of *Col1α1*, *Col3α1* and *Fn1* (Table 2, Figure 8). In comparison, diabetic *MR^My^* mice reduced the deposition of total collagen and collagen 1 in diabetic hearts and also reduced the gene expression of *Ctgf*, suggesting protection from diabetes-induced cardiac fibrosis (Figure 8).

**Figure 9:**
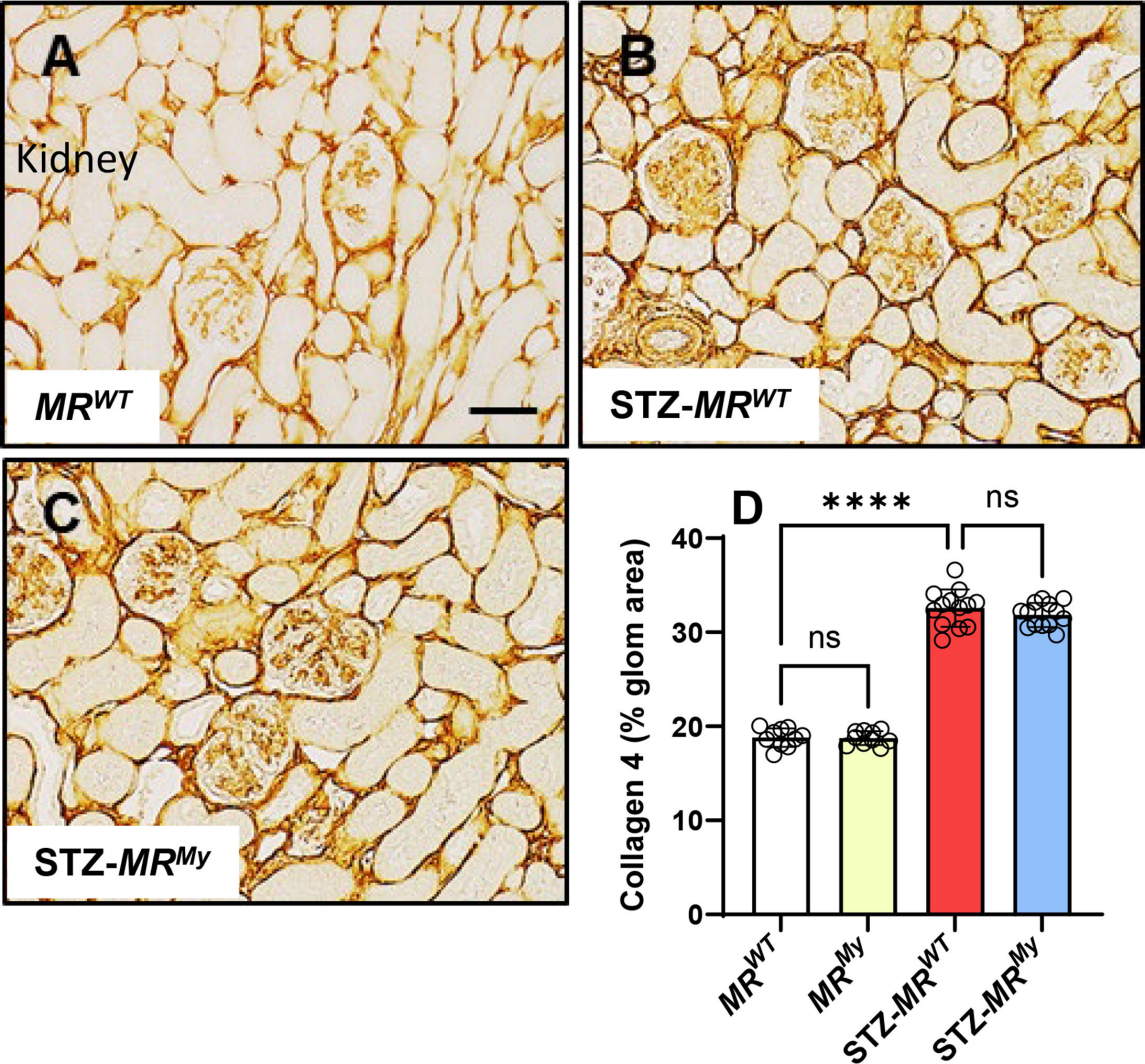
Deficiency of myeloid MR signalling does not reduce diabetic glomerulosclerosis. Immunostaining of collagen type IV (brown) is present in a small portion of the glomerulus in (**A**) a non-diabetic *MR^WT^* mouse, but is substantially increased in (**B**) a diabetic *MR^WT^* mouse due to the development of glomerulosclerosis, which is similar in (**C**) a diabetic *MR^My^* mouse. Graphical analysis shows (**D**) the percentage glomerular area immunostained with collagen IV antibody in experimental mice. Images: Bar (**A-C**) = 50 µm. Graph displays individual data points with mean ± SD, *n* = 10–13. ****P<0.0001, ns = non-significant.

Diabetic mice also displayed significant kidney injury at week 15. Diabetes increased kidney expression of genes associated with injury (*Kim1*, ten-fold), senescence (*Cdkn1a*, *Cdkn2a,* 10 to 18-fold) and fibrosis (*Tgfb1, Ctgf, Pai1, Timp1, Col1α1*, *Col4α1* and *Fn1*, two- to six-fold) (Table 1). The kidney expression levels of these genes were unaffected by deficiency of myeloid MR signalling. In comparison, kidney gene expression of *Vegfa* and *Pdgfb* did not change in *MR^WT^* mice with diabetes (Table 1). However, myeloid MR deficiency did increase *Vegfa* levels in diabetic kidneys by 44% compared with diabetic *MR^WT^* mice (Table 1). Diabetic kidneys also exhibited glomerulosclerosis, which was identified by glomerular accumulation of collagen 4. In *MR^WT^* mice, the glomerular area of collagen 4 deposition increased from 19% to 33% with the development of diabetes (Figure 9). This development of glomerulosclerosis was similar in diabetic *MR^My^* mice.

## Discussion

Our animal model study demonstrates that MR signalling in myeloid cells plays a significant role in cardiac and kidney function impairment during the development of type 1 diabetes without having any impact on established hypertension. In these organs, deficiency of myeloid MR signalling reduced macrophage accumulation and promoted the development of an M2 anti-inflammatory macrophage phenotype during diabetes, which led to specific effects on vascularisation, hypertrophy and fibrosis. The impact of these effects was more profound in the hearts than in the kidneys of these diabetic hypertensive mice.

Although MR deletion in myeloid cells affects both macrophages and neutrophils, current evidence suggests that the impact of this deletion is mainly through effects on macrophages as these cells are vastly more abundant in diabetic tissues and have an established role in the pathology of diabetic tissue injury [18,19]. Compared with non-diabetic mice, macrophage accumulation was substantially greater in diabetic kidneys compared with the diabetic hearts of *MR^WT^* mice, suggesting that the mechanisms underlying this increased macrophage accumulation may be organ specific. Notably, the increased accumulation of CD68+macrophages in diabetic hearts and glomeruli was dependent on myeloid MR signalling, whereas the accumulation of interstitial macrophages in diabetic kidneys was not. Despite these differences, macrophages in both the heart and kidneys of diabetic mice showed distinct changes towards an M2 anti-inflammatory phenotype in the absence of myeloid MR signalling, which included up-regulation of *Il4rα* and *Cd163*. Similar phenotype changes have previously been reported in other mouse models of non-diabetic heart and kidney disease where myeloid MR signalling is absent [10,13,14], suggesting that myeloid MR signalling is important in determining macrophage phenotype and responses across a range of cardiorenal diseases.

Myeloid MR signalling appears to impair cardiac and renal function through different mechanisms. In diabetic hearts, deficiency of myeloid MR signalling inhibited gene expression of a marker of cardiac hypertrophy (*Myh7*) and reduced cardiomyocyte size [20] while preventing loss of capillaries and expression of genes required for vascularisation (*Vegfa* and *Pdgfb*) [21–23], which suggests a role for myeloid MR signalling in diabetic cardiomyopathy. Furthermore, myeloid MR deficiency inhibited cardiac gene expression of *Ctgf* and also reduced accumulation of collagen in diabetic hearts based on both histological and immunostaining techniques, suggesting that myeloid MR signalling contributes to cardiac stiffness during diabetes by promoting cardiac fibrosis [24], which is consistent with previous studies of non-diabetic MR-dependent cardiac disease [11,13]. In comparison, kidney gene expression of *Vegfa* and *Pdgfb* was not altered by the development of diabetes in myeloid MR intact mice, but myeloid MR deficiency increased kidney expression of *Vegfa* during diabetes. In addition, myeloid MR deficiency did not affect the elevated expression of profibrotic genes (*Tgfb1, Ctgf, Col1α1* and *Col4α1)* or increased glomerular deposition of collagen 4 in diabetic kidneys. Therefore, the ability of myeloid MR signalling to promote renal function impairment may be dependent on its capacity to promote glomerular accumulation and induce a M1-phenotype in these macrophages which subsequently restricts glomerular filtration.

Vascular-modulating cytokines are critical for cardiac health. VEGF-A induces myocardial angiogenesis and enhances vascular permeability and endothelial cell proliferation [21,25]. In comparison, PDGF-B is essential to stabilise newly formed blood vessels and to prevent vessel regression [23], but can also promote cardiac fibrosis [26]. Consequently, a lack of VEGF-A and PDGF-B, or significant overproduction of these growth factors, can impair capillary maintenance and cardiac function. Indeed, cardiomyopathy is associated with lower production of VEGF-A and cardiac dysfunction [22,27]. In hearts, VEGF-A is mainly produced by cardiomyocytes but can also be produced by macrophages and endothelial cells [25,27]. During diabetes, myeloid MR signalling promotes macrophage accumulation and an M1 phenotype in cardiac macrophages [13]. In contrast, *MR^My^* mice develop more M2 macrophages during diabetes, which have elevated VEGF production [28] and may also stimulate cardiomyocyte production of VEGF-A by producing TGF-beta [13]. Thus, our findings indicate that deficiency of myeloid MR signalling restores production of VEGF-A and PDGF-B which helps to prevent capillary loss and cardiac dysfunction.

Diabetes-induced kidney senescence may partly explain why myeloid MR deficiency protects hearts more than kidneys during diabetes. Approximately half of all kidney cells are proximal tubular epithelial cells, and many of them become senescent in response to diabetes. Senescent tubular epithelial cells (TEC) are known to regulate kidney inflammation and fibrosis [29]. In our study, the development of diabetic kidney cell senescence was indicated by a marked increase in gene expression of *Cdkn1a* and *Cdkn2a*, which was unaffected by myeloid MR deficiency. In contrast, diabetic hearts contain only a minor component of epithelial cells (in valves, vessel linings and epicardium) and the majority of cardiac cells (myocytes, endothelial cells, fibroblasts) are less susceptible to diabetes-induced senescence, as indicated by a lack of increase in *Cdkn1a* and *Cdkn2a* gene expression in diabetic hearts. Therefore, it is feasible that the activity of infiltrating macrophages may be more strongly affected by myeloid MR deficiency in diabetic hearts than in diabetic kidneys, due to the relative differences in impact of cellular senescence.

Previous studies have shown that myeloid MR signalling is important in the development of cardiac fibrosis in non-diabetic models of hypertension, in which hypertension is reduced [9,10,13]. In contrast, our study shows that myeloid MR signalling is also important for diabetes-induced cardiac fibrosis in the absence of any effect on pre-existing hypertension. This finding is also supported by non-diabetic models of aldosterone-independent and angiotensin 1 receptor-independent cardiac injury showing that myeloid MR signalling can promote cardiac fibrosis in the absence of an effect on hypertension [11,30]. Therefore, it appears that diabetes induces cardiorenal dysfunction by directly regulating tissue inflammation and fibrosis via myeloid MR signalling.

Our findings have significant implications for the use of MRAs in diabetic patients with cardiac and kidney disease. Indeed, we have shown that selective targeting of MR in myeloid cells can reduce the macrophage-mediated injury and tissue remodelling responses that are induced by diabetes, resulting in protection of organ function. This targeting approach, which is independent of an effect on hypertension, provides greater protection for the heart than kidneys during diabetes, suggesting that the pathological effects of myeloid MR signalling are more critical to the development of cardiac disease than kidney disease during diabetes. Our outcomes suggest that selective delivery of MRAs to macrophages may be an effective way to reduce diabetic cardiorenal injury without the risk of causing hyperkalaemia. However, it is noteworthy that MR signalling in other cardiac cells (endothelial cells, vascular smooth muscle cells and cardiomyocytes) has also been shown to promote cardiac damage in models of non-diabetic cardiac injury [31]. Therefore, optimal protection against cardiorenal injury in diabetes may require targeting MR signalling in multiple cell types.

**Clinical perspectives**

- Clinical research indicates that treatment with mineralocorticoid receptor antagonists (MRAs) can suppress the development of cardiac and kidney injury in patients with diabetes and hypertension but is limited by their ability to induce hyperkalaemia.
- Using a novel transgenic mouse model, we have identified that genetically deleting MR signalling in myeloid cells can protect against cardiac and kidney dysfunction caused by diabetes and hypertension.
- This study indicates that selective delivery of MRAs to macrophages has the potential to reduce cardiorenal injury in diabetic patients without the risk of inducing hyperkalaemia.

## Supplementary material

online supplementary table 1.

Uncited online supplementary figure 1.

## Data Availability

All authors agree to make any materials, data and associated protocols available upon request. Data from individual samples are displayed in most figures.

## Abbreviations

CKD: chronic kidney disease
CVD: cardiovascular disease
MRAs: mineralocorticoid receptor antagonists
PLP: paraformaldehyde–lysine–periodate
SBP: systolic blood pressure
STZ: streptozotocin
