# Supplementary material for: Deficiency of mineralocorticoid receptor signalling in myeloid cells protects cardiac and kidney function in hypertensive diabetic mice
Source: Clin Sci (Lond). 2025 Dec 19;139(24):1675–89. doi: 10.1042/CS20256132 (PMC12794314; doi:10.1042/CS20256132)
Supplement: online supplementary table 1. [file cs-139-24-CS20256132-s001.docx]

**Supplementary Table 1 : Genotyping PCR primer sequences**

The presence of the MR wild type and MR floxed alleles, and the Cre transgene were determined by PCR analysis of genomic DNA from tail tips.

| **Target** | **Forward Primers** | **Reverse Primers** |
| --- | --- | --- |
| **MR** | TTCTTTCCCCAGCTCTACCTTTACGA | AGCAAGAGACAACTGCAGCGTTTTA  ATGTGGAATGTGTGCGAGGCCAGAG |
| **Cre** | ATGTCCAATTTACTGACCG | TTCACTGGTTATGCGGCG |

**Supplementary Table 2: Commercial mouse gene expression assays**

(Applied Biosystems, Thermo-Fisher Scientific, USA )

| **Target gene** | **Taqman™ Gene**  **Expression Assay ID** |
| --- | --- |
| *Β-Mhc* | Mm00600555_m1 |
| *Kim-1* | Mm00506686_m1 |
| *Cdkn1a* | Mm00802529_m1 |
| *Cdkn2a* | Mm00494449_m1 |
| *Elane* | Mm00469310_m1 |
| *Adgre-1* | Mm00802529_m1 |
| *Cd68* | Mm03047343_m1 |
| *Ccl-2* | Mm00441242_m1 |
| *Tnf-α* | Mm00443258_m1 |
| *Nos-2* | Mm00440502_m1 |
| *Nox-4* | Mm00479246_m1 |
| *Nlrp-3* | Mm00840904_m1 |
| *Arginase-1* | Mm0475899_m1 |
| *Cd163* | Mm00474091_m1 |
| *Cd206* | Mm01329362_m1 |
| *IL-4ra* | Mm01275139_m1 |
| *IL-10* | Mm01288386_m1 |
| *Vegf-α* | Mm01281449_m1 |
| *Pdgf-β* | Mm00440677_m1 |
| *Tgf-β1* | Mm01178820_m1 |
| *Ctgf* | Mm01192933_m1 |
| *Timp-1* | Mm01341361_m1 |
| *Collagen-1α1* | Mm00801666_g1 |
| *Collagen-3α1* | Mm01254476_m1 |
| *Collagen-4α1* | Mm01210125_m1 |
| *Fibronectin* | Mm01256744_m1 |

**Supplementary Table 3: Cardiac Echo Data**

|  | **Mouse Treatment and Genotype** | | | |
| --- | --- | --- | --- | --- |
| **Parameter** | **ND-MR^WT^** | **ND-MR^My^** | **STZ-MR^WT^** | **STZ-MR^My^** |
| n | 10 | 10 | 13 | 13 |
| LVIDd, mm | 3.1 ± 0.07 | 3.1 ± 0.08 | 2.8 ± 0.17^a^ | 3.0 ± 0.13^b^ |
| LVIDs, mm | 2.2 ± 0.09 | 2.2 ± 0.08 | 2.2 ± 0.08 | 2.1 ± 0.10 |
| FS (%) | 29.1 ± 4.1 | 29.9 ± 3.5 | 23.6 ± 4.7^a,c^ | 29.0 ± 4.5 |

ND = non-diabetic

STZ = streptozotocin-induced diabetes

MR^WT^ = mineralocorticoid receptor wild type

MR^My^ = mineralocorticoid receptor deficiency in myeloid cells

n = sample number

LVIDd = left ventricular internal diameter diastolic

LVIDs = Left Ventricular Internal Diameter systolic

FS = fractional shortening

^a^p<0.0001 vs ND-MR^WT^, ^b^p<0.01 vs ND-MR^My^, ^c^p<0.05 vs STZ-MR^My^
