# Supplementary material for: Deficiency of mineralocorticoid receptor signalling in myeloid cells protects cardiac and kidney function in hypertensive diabetic mice
Source: Clin Sci (Lond). 2025 Dec 19;139(24):1675–89. doi: 10.1042/CS20256132 (PMC12794314; doi:10.1042/CS20256132)
Supplement: Uncited online supplementary figure 1. [file cs-139-24-CS20256132-s001.pdf]

Supplementary Figure 1: Summary of Findings

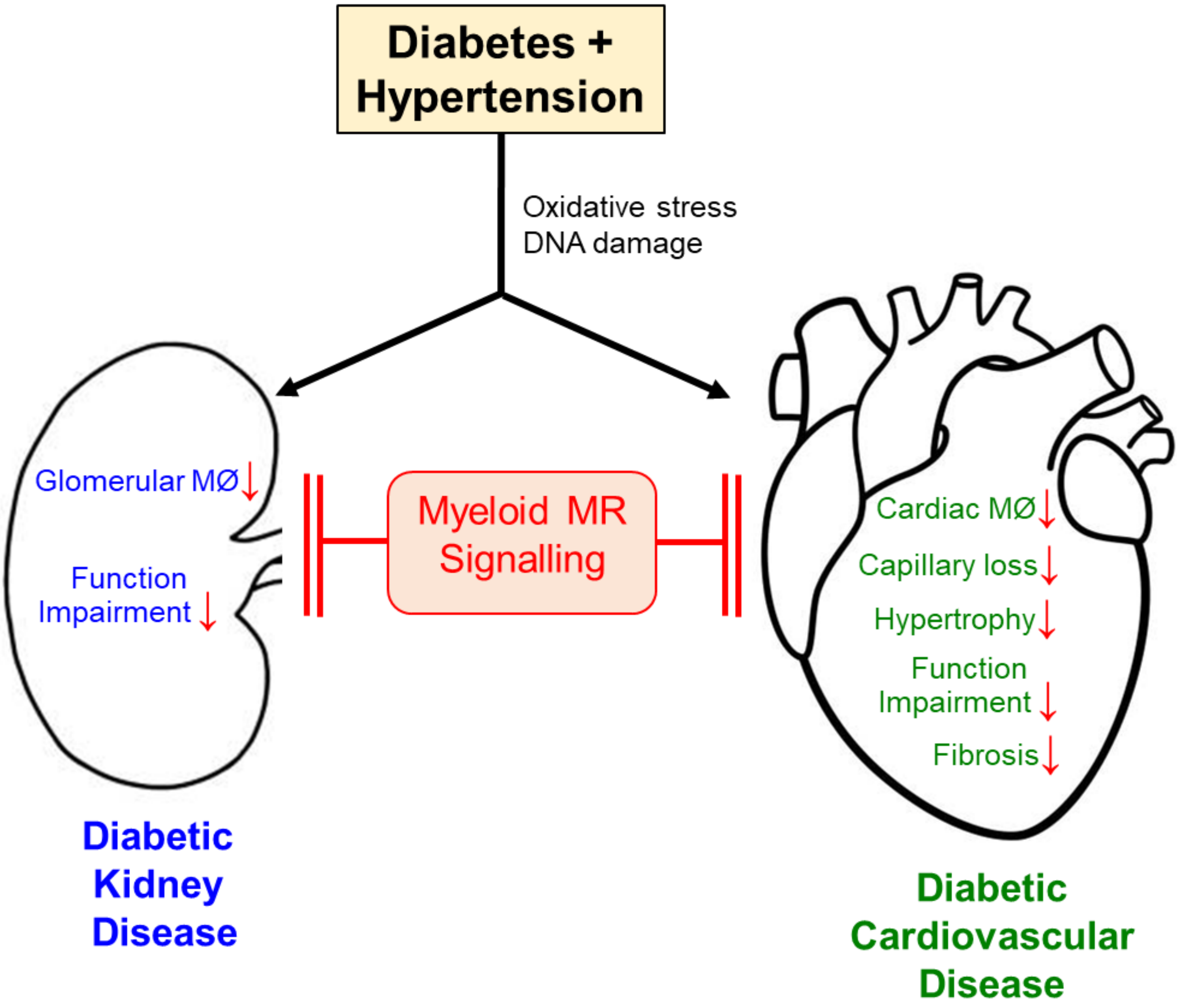

Suppl. Fig. 1: Impact of myeloid MR signalling on kidneys and heart during diabetes and hypertension. Diabetes and hypertension induce oxidative stress and DNA damage causing the development of diabetic kidney disease and diabetic cardiovascular disease. Genetic deficiency of myeloid mineralocorticoid receptor prevents myeloid MR signalling which protects these organs from diabetic injury. In diabetic kidneys, this protection is only mild, resulting in reduced numbers of glomerular macrophages (MØ) and partially diminished renal function impairment. In contrast, the protect seen in diabetic hearts is more profound, resulting in reductions in cardiac macrophage accumulation (MØ), capillary loss, hypertrophy, fibrosis and cardiac function impairment.
